# 

**Published:** 1849-07

**Authors:** 


					LIBRARY.
A Treatise on the Diseases and Surgical Operations of the Mouth,
and Parts icent Translated from the French of M. Jour-
dam, by P. H. Austen, A. M>
E .? I
5,.-
Bv William H. Dwindle, M. D? D. p. S.,
Article &
m Chirtes H. Dubs' P&amt Compound Union
JW Forceps. Dr. S. P. Hallihen'a Root Forceps,
335 I
Article II.?
Extract of a Letter to the Baltimore Editor, from
I Mr. Aadrew Wilson, of Edinburgh,
Artict* IIL?-
aud preTioasiy to the Death or Destruction of the Pulp.
Chapin A. Harris, Nt D.
340
Article IV.?
Treatment of Dental Caries, when the Cavity extends
nearly to the Nervous Pulp,
By G. 0. Cone, M. D., of Bait.,
Article v.-
-Remarks on the Use of Clasps for tha Retention of
Artificial Teeth.
By P. H. Austen, M. D., Baltimore,
357
Dental Education.
By W..R. Handy, M. D.,
BIBLIOGRAPHICAL NOTICES.
?
*3
Soow, .
Portraits of the Skin,
m-':
by Erasmus Wilson, F. R. S., Fasciculus,
An Essay on the Teeth,
by A. Cook,
Notice upon some of the Diseases of the Mouth, &c.
Br Hattute,
Chemical Analysis, Qualitative and Giuantitaave,
by Henry M.
fk IWoad'L,ectureron~v
MISCELLANEOUS NOTICES.
Postponement of the Tenth Annual Meeting of the American Souv*y
of Dental Surgeons,
m fj
Specimens of Morbid Dental Anatomy,
V
Mr. Evana* Amalgam for filling Teeth,
Malformation of a Lower Incisor,
m A New Operating Chair,
r. t- msasm
Baltimore College of Dental Surgery,
Mr. Clark's Patent Method of Inserting Pivot Teeth,
378
Jk h
v
The Library Part of the Journal^
The Late Dr. Alexander Nasmyth,
imposition Practiced by Dentists,
Artificial Teeth Mounted on Plate, adapted to the Mouth over Dis- i
eased Roots,
. f C t
Professional Items,
A Charge of Professional Piracy,
To Correspondents,
.
?Tr\.
To Agents and Subscribers,
.
i - r
Obituary,
.
COLLE CTANEA.
Strictures on the Destruction of the Nerves in the Treatment of Ca- ?:
Teeth,
gi< s -?r
V ?
Lymphatic Vessels of the Tongue, i
. ?
Influence of the Pneumo-gastric Nerves on Digestion,
387
Indian Hemp in Facial Neuralgia,
387
* The
Homy Growth from the Head in

				

